# 

**Published:** 1866-11

**Authors:** 


### INDEX OF HALL'S HEALTH TRACTS.

**Containing 236 Health Tracts on the following subjects, with a steel engraving of the Editor, sent post-paid for $2.50.**

Aphorisms Physiolog'l.
Apples.
Antidote to Poisons,
Acre, One.
Apoplexy.
Burying Alive.
Baths and Bathing.
Beards,
Bites and Burns.
Backbone,
Beauty a Medicine.
Best Day.
Baldness.
Burning to Death.
Bilious Diarrhea.
Balm of Gilead.
Bread.
Cold Cured.
" Neglected.
" Avoided.
" Nothing but a.
" In the Head.
" How Taken.
" Catching.
Consumption.
Coffee-Drinking.
Catarrh.
Checking Perspiration.
Centenarian.
Child-Bearing.
Children's Eating.
" Feet.
Children Corrected.
" Dirty.
Coal-Fires.
Cute Things.
Coffee Poisons.
Clothing, Flannel.
" Woolen.
" Changing.
Cholera.
Clergymen.
Cancer.
Corn-Bread.
Convenient Knowledge.
Charms.
Cooking Meats.
Cheap Bread.
Church Ventilation.
Cough.
Dyspepsia.
Drinking.
Diet for the Sick.
Deafness.
Debt, a Death!
Diarrhea.
Death.
Dying Easily.
Drowning.
Diptheria.
Dysentery.
Disinfectants.
Death Rate.
Deranged.
Digestibility of Food.
Dirty Children.
Drugs and Druggery.
Eyes, Care of.
" Weak.
" Failing.
Erect Position.
Eating.
" Wisely.
Eat, How to.
" What and when to.
Eating Habits.
" Great.
" Curiosities of.
" Economical.
Emanations.
Elements of Food.
Fruits, Uses of.
Flannel Wearing.
Follies, Fifteen.
Fire-Places.
Fifth Avenue Sights.
Food and Health.
Feet.
Fetid Feet.
Food, Nutritiousness of.
" its Elements.
Greed of Gold.
Genius, Vices of.
Great Eaters.
Gruels and Soups.
Hair Wash.
Health a Duty.
" Observances.
" Essentials.
" Theories.
Hydrophobia.
Headache.
Housekeeping.
Housewifery.
Household Knowledge.
Habit.
Home, Leaving.
Happiest, Who are.
Hunger.
Inconsiderations.
Ice, Uses of.
Inverted Toe-Nail.
Insanity.
In the Mind.
Kindness Rewarded.
Law of Love.
Longevity.
Life Wasted.
Loose Bowels.
Leaving Home.
Logic Run Mad.
Medicine Taking.
Memories.
Music Healthful.
Milk, its Uses.
Miasm.
Marriage.
Morning Prayer.
Month Malign.
Mental Ailments.
Mind Lost.
Medical Science.
Neuralgia.
Nursing Hints.
Nervous Debilities.
Old Age Beautiful.
One Acre.
One by One.
Obscure Diseases.
Precautions.
Presence of Mind.
Premonitions.
Private Things.
Poisons and Antidotes.
Pain.
Peaceless.
Preserves.
Parental Trainings.
Philosophy.
Physiological Items.
Posture in Worship.
Physician, Faithless.
Popular Fallacies.
Punctuality.
Providence.
Rheumatism.
Read and Heed.
Resignation.
Restless Nights.
Recreation, Summer
Restlessness.
Sitting Erectly.
Shoes Fitting.
Sabbath.
Sour Stomach.
Sick Headache.
Sunshine.
Skating.
Suppers, Hearty.
Soldiers Remembered.
" Cared for.
" Health.
" Items.
" All.
Serenity.
Sores.
Sunday Dinners.
Small-Pox.
Sleep and Death.
Spot the One.
Specifics.
Spring-Time.
Summer Drinks.
Sickness not Causeless.
Sayre, the Banker.
September Malign.
Summer Mortality.
Stammering.
Soups and Gruels.
Sick School-Girl.
Stomach's Appeal.
Sleep.
Summerings.
Study, Where to.
Salt Rheum.
Sordid.
Traveling Hints.
Three Ps.
Teeth.
Toe-Nail, Inverted.
Thankful Ever.
Urination.
Valuable Knowledge.
Vaccination.
Ventilation.
Vermin Riddance.
Winter Rules.
Walking.
Warning Youth.
Woman's Beauty.
Whitlow.
Whitewashes.
Worth Remembering.
Worship, Public.
" Posture in.
" Without Price.
Weather Signs.
Weather and Wealth.
Warmth and Strength.
Worth Knowing.

Address "Hall's Journal of Health," No. 2 West 43d St., New-York, ($1.50 a Year.)

### GREAT INDUCEMENTS FOR 1867!

The *American Agriculturist* is published monthly for $1.50 a year; it is the cheapest, best, and most ably edited agricultural monthly on the globe; every issue has more than one hundred practical articles relative to the cultivation of fruit, flowers, grains, cattle, &c. To any person sending us $2 before the first day of December, both publications will be sent for 1867, including the December issues of each; this offer will expire on the last day of November, 1866, positively.

THE SCIENTIFIC AMERICAN.— The oldest, most successful, and ablest paper of its kind in the world; as practically useful for every household as for scientific men and inventors, and all men of progress, is published weekly at $ 3 a year. We will send it and HALL'S JOURNAL OF HEALTH for 1867, for $ 3 a year, thus giving both publications for the price of one.

THE PHRENOLOGICAL JOURNAL, now in its 43d volume, is published monthly at 389 Broadway, New York, at $ 2 a year, profusely illustrated with the portraits of eminent characters, and filled with a great variety of useful reading matter pertaining to the conduct of life, and mental moral, and social improvement. It will be furnished for 1867, with HALL'S JOURNAL OF HEALTH, for $ 2.50, thus affording our Journal for 50 cents a year.

THE NEW YORK INDEPENDENT is published weekly at $ 2.50 a year. It is written for by some of the ablest minds in the nation; it has weekly letters from well informed correspondents abroad, and gives in every issue copious monetary and market reports, prepared with great accuracy and industry; we offer it and the JOURNAL OF HEALTH, for 1867, for $ 3 to every person who has not taken the *Independent* before.

These offers will be all withdrawn on the last day of the year 1866.— Those who send the money to *Hall's Journal of Health*, No. 2 West 43d St., New York, previous to the 1st day of December, 1866, will have a December number of each publication subscribed for, without charge.

Five copies of *Hall's Journal of Health* for 1867 will be sent to one address for $ 5; to Clergymen and Theological students of all denominations, the *Journal of Health* will be sent for 1867 for $ 1.

To any fifty Foreign Missionaries, who first apply, the *Journal* will be sent free, for 1867, free of postage. We wish we felt able to send it to all of them, for they are doing more to raise the world from barbarism to civilization, refinement and religion, than a thousand times their number of princes, potentates, statesmen and kings, while they work, and work hard, self-expatriated from their native land, for nothing more than their food and clothing, and these of a pretty plain kind, for the most part.

